# First Step toward Gestural Recognition in Harsh Environments

**DOI:** 10.3390/s21123997

**Published:** 2021-06-09

**Authors:** Omri Alon, Sharon Rabinovich, Chana Fyodorov, Jessica R. Cauchard

**Affiliations:** 1 Magic Lab, Department of Industrial Engineering and Management, Ben Gurion University of the Negev, P.O. Box 653, Beer-Sheva 8410501, Israel; 2 Autonomous Systems Lab, University of California, Santa Cruz, CA 95064, USA; srabinov@ucsc.edu; 3 Department of Asian Studies, Faculty of Humanities, Haifa University, Haifa 3498838, Israel; saardita@gmail.com

**Keywords:** harsh environments, gesture recognition, first responders, firefighting, remote sensing, HRI, drone, robot

## Abstract

We are witnessing a rise in the use of ground and aerial robots in first response missions. These robots provide novel opportunities to support first responders and lower the risk to people’s lives. As these robots become increasingly autonomous, researchers are seeking ways to enable natural communication strategies between robots and first responders, such as using gestural interaction. First response work often takes place in harsh environments, which hold unique challenges for gesture sensing and recognition, including in low-visibility environments, making the gestural interaction non-trivial. As such, an adequate choice of sensors and algorithms needs to be made to support gestural recognition in harsh environments. In this work, we compare the performances of three common types of remote sensors, namely RGB, depth, and thermal cameras, using various algorithms, in simulated harsh environments. Our results show 90 to 96% recognition accuracy (respectively with or without smoke) with the use of protective equipment. This work provides future researchers with clear data points to support them in their choice of sensors and algorithms for gestural interaction with robots in harsh environments.

## 1. Introduction

Mobile ground and aerial robots (also known as drones) offer unique potential to support people in first response situations, such as in firefighting, search and rescue, police, and military situations. These robots are now sent to explore areas, perform tasks with or instead of humans, and thus reduce the risk to people’s lives. Recently, we have been seeing a technological shift, with robots presenting increasing levels of autonomy [1,2,3], meaning that they can now directly interact with first responders. Such technological advances have led human–computer and human–robot interaction (HCI/HRI) researchers to explore natural ways for first responders to communicate with these robots, with one of the most prevalent ways being gestural interaction (e.g., using finger, hand, or full-body gestures to command and instruct a robotic agent) [4,5,6]. However, the conditions experienced by first responders in their missions present non-trivial challenges for gestural interaction sensing and recognition, such as when working in low visibility (e.g., in smoke or darkness). These situations are referred to as “harsh environments”, which further require first responders to wear heavy protective equipment, presenting yet additional constraints for gesture sensing and recognition.

While there is a large body of prior work on gestural interaction, including sensing techniques and algorithms for recognition, the literature about gestural recognition in harsh environments is sparse. Research is needed to understand which sensors and algorithms are best suited to support gestural interaction in harsh environments. In this manuscript, we build upon the preliminary work conducted by De Cillis et al. [7], which investigated the use of a depth camera to sense three types of gestures that were then trained and tested with two people (one and one) in simulated harsh conditions. We take their approach further and compare a depth camera with two different types of visual sensors: RGB and thermal cameras. We also investigate a range of algorithms to enable gesture recognition with each of the three sensors, using user-defined gestures that were specifically chosen by operational firefighters for interacting with drones in firefighting missions. We trained our system with 13 people and tested it with two people in several conditions, including simulated harsh environments. This work provides a review of possible technological solutions that can be embedded in ground robots and drones for gesture recognition in harsh environments, including the advantages and drawbacks of the different sensors and algorithms.

In this manuscript, we first present a literature review and then describe a set of gestures adapted to human–drone interaction (HDI) in firefighting. We then present our implementation of a gesture recognition system, based on a range of algorithms, which are then tested with the three different types of sensors. We present the system’s evaluation in laboratory settings, simulating different conditions of harsh environments. Our results show major differences between the three types of sensors regarding the recognition accuracy for different simulated environments. For example, we found that while the overall recognition accuracy dropped under smokey conditions, the accuracy stayed high for RGB and thermal cameras, while the depth sensor failed to recognize gestures. Our results present opportunities for future research on remote sensing in harsh environments and inform future HCI and HRI researchers and practitioners on the human and technical aspects to consider when integrating gestural recognition systems in emergency responses. This work contributes to a larger understanding of gestural recognition in harsh environments, as well as a comparison between three common sensors and various algorithms that can be used for remote sensing in harsh environments.

## 2. Related Work

In the following section, we review the related prior work, first on ground and aerial robots, then on gestural recognition, and finally on remote sensors, all in harsh environments.

### 2.1. Ground and Aerial Robots in Harsh Environments

“Harsh environment” is a broad term that can refer to “any environment that is hazardous to agents (human or robot, etc.) within it. For example, they can be characterized by high levels of radiation, high explosive risk, extreme temperatures or pressures, and lack of oxygen” [8]. Harsh environments can also be defined as environments that are “unknown, unstructured, dynamic, cluttered, hazardous and/or limited in resources (such as the availability of communications, GPS and visibility)” [9]. These environments present unique challenges for integrating robots, such as open fire sources, dynamic obstacles, and faulty sensor readings [10]. In particular, harsh environments present many challenges for the remote sensing community, with issues such as low visibility due to smoke or darkness, which can disturb visual sensing, or noise, which can prevent audio sensing. Furthermore, such environments present additional challenges for controlling robots and drones—for example, when GPS signals might be unavailable—or even for a user to directly interact with the robotic agent while under pressure, with a high workload, and while potentially wearing heavy protective equipment.

The HCI and HRI communities have investigated and proposed technological solutions for drones and robots to support first responders in their missions, in harsh environments, such as in firefighting [11], search and rescue [12], police [13] work, and in the military [14]. In particular, in firefighting, drones and robots have been used for a variety of applications, such as searching for victims and checking the environment and surroundings in search and rescue missions [15], detecting and extinguishing fires [16,17], and also in mapping and detecting gas in both indoor and outdoor environments [18,19]. A recent report summarized the benefits of using drones for firefighting missions, including visual access to low-visibility areas, increased safety for firefighters during emergency missions, optimized data gathering processes, time-saving, and reduction of human error in inspections of a zone [20].

As autonomous drones and robots emerge [2,21], researchers are interested in the transition of these robots from a tool to a teammate [22,23]. This paradigm shift drives the need for collocated interaction strategies between robots and humans [24], with one of the best strategies for such collocated interaction being gestures [25,26,27]. In this work, we propose to explore remote sensing and algorithms for gestural interaction with robots in harsh environments, using the example of firefighting missions given the amount of prior supportive literature from the fields of HCI and HRI. The following subsection describes the current state-of-the-art for gestural interaction in harsh environments.

### 2.2. Gestural Recognition in Harsh Environments

Gestural interaction in harsh environments holds major constraints for sensing visual stimuli and making sense of the information. Indeed, low-visibility conditions (e.g., smoke, darkness) can disturb the sensing. In addition, humans interacting with the system may be wearing heavy protective equipment, so that finger, hand, and body tracking may not be achieved with regular image segmentation (e.g., skeleton extraction). The existing body of work on gestural recognition in harsh environments is sparse. After extensively surveying the literature for first responders, we only found one prior work investigating supporting firefighters with robots using gestures [7]. The work proposed using a depth camera to sense three types of gestures (simple, medium to challenging, and hard gestures) that were then tested in simulated harsh conditions in a lab (i.e., darkness and smoke, indoor and with uniform). Unfortunately, while the work contributed a framework for gesture recognition in HRI, the manuscript did not adequately report on the gestures that were implemented, their suitability for firefighting, the conditions of the evaluation, or the exact simulated environment. Therefore, further research is needed to investigate which sensors and algorithms are most appropriate in harsh conditions and to propose adapted settings for laboratory simulation of harsh environments.

To conduct this research, we further report on the large body of literature on gestural recognition and remote sensing, focusing on the types of gestures and the recognition algorithms. Gestural interaction can be based on various body parts, from full-body [28], to arm [29], hand, and foot gestures [30], and even finger movements [31]. Escalera et al. [32] created a taxonomy for the various components involved in conducting research in gesture recognition and proposed that two categories of gestures can be distinguished: 1. *Static* gestures that represent a still pose in a single image, and 2. *Dynamic* gestures are represented by a sequence of images. Gestural interaction distinguishes between gestures that give commands to an agent and gestures that are meant to initialize or terminate the interaction, as is the case in our work. For example, prior work used the familiar arm-waving gesture as a signal to attract a UAV’s attention [26,33]. In our work, we propose to use a set of gestures that were elicited following a user-centered design process with firefighters [23].

In terms of interaction, many prior works investigated gestural interaction systems with computers, robots, and drones [34,35,36,37]. The literature offers a variety of approaches for gestural recognition, which are described in a recent survey [38], where the authors categorized algorithms into three categories: hand-crafted motion features, deep learning-based methods, and depth information-based methods. It is, however, clear from their work that the different methods are not equivalent and hold advantages and drawbacks based on the types of gestures to recognize, situations, and chosen sensors. As firefighting scenes may have smoke and generally low visibility, they need special care with regard to the choice of sensor. In the following section, we discuss sensors for remote gesture sensing in harsh environments.

### 2.3. Remote Sensing in Harsh Environments

Different types of visual sensors exist, each relying on different technologies, working across a range of the light spectrum. As such, different sensor types will perform differently in harsh environments, some working in daylight, others in darkness, or even able to sense the environmental temperature. In their work, Berman and Stern [39] created a taxonomy for sensors for gesture recognition systems and showed that optical sensors are traditionally used for recognizing gestures. Three main types of optical sensors are used in the literature [32]: RGB [40], thermal [41], and depth [42] sensors. In harsh environments, only a few works have compared sensors, such as Starr and Lattimer [43], who evaluated seven different types of sensors in fire and smoke conditions—in terms of performance, but not for gesture sensing. They divided their experiment into two categories of harsh environments: 1. light smoke with high temperature, and 2. dense smoke with low temperature. In the former, RGB and thermal sensors worked but the depth camera failed, and in the latter, the RGB sensor failed below 1 m visibility and encountered attenuation past 8 m visibility; the depth sensor showed poor results even with a visibility over 8 m, while the thermal sensor was not affected by the conditions. These results suggest that a thermal sensor may be the best choice for a gestural interaction system in firefighting, where thick smoke and darkness can be expected, and that RGB and depth sensors will be more likely to fail when smoke is present.

In our work, we propose to compare RGB, depth, and thermal sensors that can later be embedded into a drone or robot, and to evaluate their performance for gesture recognition in simulated firefighting conditions. We expect that the heavy protective equipment and smoke conditions will decrease the recognition accuracy, compared to optimal conditions. Furthermore, we will investigate how the simulated harsh environment conditions will affect the various sensors and algorithms that we implement. The next section describes the chosen set of gestures.

## 3. Gestures for Gestural Communication in Harsh Environments

In this section, we describe our choice of gestures to be implemented and evaluated. We opted for gestures that were previously elicited in a user study with firefighters [23], which focused on gestures for collocated interaction with a drone. We first describe the tasks allocated to the drone, as identified by the firefighters, and their matching gestures to instruct the drone. The four main drone tasks and associated user-defined gestures, as described in [23], are presented below:

- **Mapping.** The firefighters envisioned sending the drone to map a floor or even an entire building, finding out how many rooms are inside a building, what the structure of the building looks like, and creating a map of it.
- **Identifying Hazardous Materials.** The firefighters proposed that the drone could identify the type of hazardous material (such as gas or chemicals) and the source and size of the leak, which is a scenario that they encounter in industrial buildings.
- **Detecting Fires.** The firefighters proposed that the drone could identify fire spots. This is in line with current drone usage in the country.
- **Finding Survivors.** The firefighters suggested sending the drone to search for trapped civilians or people otherwise in danger. They would like the drone to gather information on the number of people as well as their condition and location. They proposed that the drone could directly provide help to citizens who have fallen into holes or deep tunnels. The firefighters further mentioned a situation that they referred to as “fear for human life and aid to civilians”. This corresponds to situations where there is a concern for civilians, who are locked in their homes and are not responding to the door, who usually have not been seen by their relatives or neighbors, who called the firefighters to break in. Participants mentioned that a drone could fly inside an apartment through a window to help them to conclude on whether they need to break into the apartment or not.

**Figure 1:**
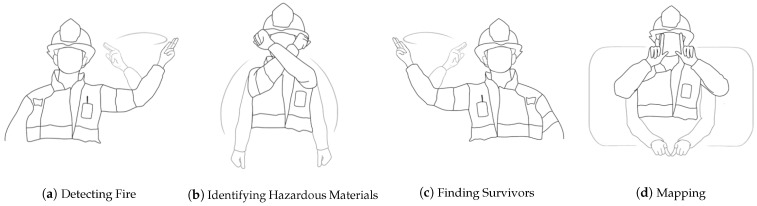
Set of four user-defined gestures used to support the following tasks: (**a**) detection of fire spots (their number and location), (**b**) identification of gas/chemical leak source and size, (**c**) search for trapped citizens or people in danger and collection of information on their number and location, (**d**) mapping of the floor or building. Image courtesy of [23].

The drone tasks are summarized in Table 1, and the elicited gestures chosen for each task (see Figure 1) include: *Detecting Fire:* lifting the left hand and rotating it clockwise with two fingers up; *Identifying hazardous materials:* raising two hands and crossing them in front of the face; *Finding survivors:* lifting the right hand and rotating it clockwise with two fingers up (same as Detecting Fire but with the right hand); *Mapping:* drawing a “frame” in front of the body using both hands symmetrically with two fingers pointing up. The firefighters chose gestures that are continuous and repeated, meaning that they will be performed until the desired command is detected (i.e., when they receive feedback that the gesture was properly understood).

Based on the physical load of the equipment that firefighters carry, one could expect the resulting gestures to be one-handed, small, and fast, saving firefighters from any additional physical and cognitive efforts to communicate with the drone. However, Alon et al. [23] revealed that, regardless of the constraints, the firefighters preferred large, noticeable, and continuous gestures, some of them two-handed. They further highlighted that these gestures are consistent with those of other first responders and military environments [44,45]. We are thus confident that these chosen gestures are representative of wider first response environments.

## 4. Gesture Recognition System Prototyping

We designed and implemented a gesture recognition system prototype for the user-defined gestures shown in Figure 1. In addition to the set of gestures, we trained the system to recognize if a person is present in front of the camera and whether they are walking or standing still. Our goal was to survey which technology is best suited for gesture recognition in harsh conditions. In this section, we first present the implemented sensors and algorithms that were used in our experiment. We then describe the data collection and training of the system, and finally, we describe the evaluation process.

### 4.1. Visual Sensors

**Figure 2:**
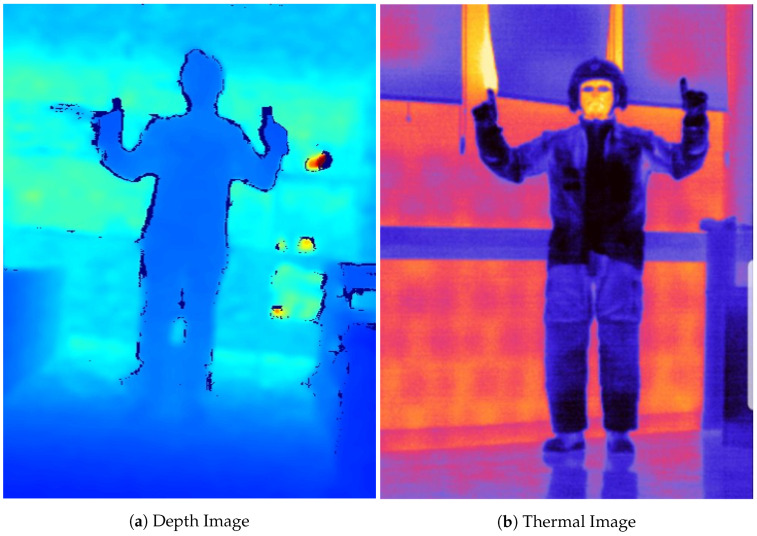
Example of images acquired in the evaluation phase. (**a**) Depth image of a person wearing a normal outfit. (**b**) Thermal image of a person wearing a firefighter’s protective equipment.

In terms of hardware for image acquisition, we compared three types of sensors, namely RGB, depth, and thermal cameras, as these are the most widespread sensors in the gestural recognition literature (e.g., RGB [46], depth [42], and thermal [47]). We used an Intel Realsense D435 [48] camera for RGB (frame rate: 30 Hz, resolution: 1920 × 1080, field of view: 69° × 42° (H × V)) and depth (frame rate: 90 Hz, resolution: 1280 × 720, field of view: 87° × 58° (H × V)) images, and a Therm-App HZ [49] for the thermal image (frame rate: 25 Hz, resolution: 384 × 288, field of view: 55° × 41° (H × V)). Figure 2 shows resulting frames from the depth and thermal sensors.

### 4.2. Algorithms

Before a system can be implemented in a robot or a drone, it needs to be validated in static conditions [37,50]. We apply this strategy to our work and present the system in static conditions. We empirically determined that the longest duration for a gesture took an average of 3 s to perform (see Section 4.3), and as such, we sampled data at a rate of 20 frames every 3 s (i.e., 6.7 Hz). The prediction was based upon the changes within the series of frames extracted in the 3 s window. We then implemented three algorithms, which we further describe below. The first one is based on skeleton extraction and the second one on a histogram of oriented gradient (HOG). Both algorithms use support vector machine (SVM) for the classification as this supervised learning model is widely used in the gesture recognition literature, both with skeleton features [51] and with HOG [52], and as it is suitable for high-dimensional representation. For the SVM, we used the scikit-learn API in Python, with linear kernel and C (regularization parameter) equal to 10. The third algorithm consists of a bespoke algorithm, “Frame Vote”, that we developed, and which can use either HOG and SVM or convolutional neural networks (CNN). Our intention was to compare several approaches to the traditional classification method of HOG + SVM often used in UAVs [53].

**Figure 3:**
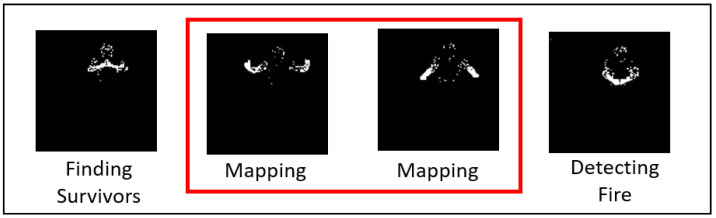
Description of the bespoke “Frame Vote” algorithm. Each frame is classified as one of the possible gestures. The final classification is made by comparing which gesture was most often classified within 20 frames. In this example, two out of four frames have been classified as “Mapping”, so the algorithm chooses that gesture over the other two.

- **Skeleton Extraction**. Key points of the person are extracted using the Realsense SDK (Cubemos), which provides a vector of 18 (x,y) coordinates for each frame. Each gesture consists of 20 frames which form a vector of 720 values (20 frames × 18 coordinates × 2 (x,y) values). Skeleton extraction is a known approach in the literature for extracting features for body gesture recognition (e.g., [54]) and it can be implemented with various APIs, such as OpenPose [55]. Compared to other feature extraction techniques, such as using 3D CNNs, which are time-consuming and may be difficult to train, the features of the human skeleton are concise and are based on a pre-trained CNN [54].
- **Histogram of oriented gradient (HOG)**. Each frame is represented by a HOG [56], so that each gesture forms a vector of 20 concatenated HOG vectors. We used scikit-image for extracting the HOG features with the number of pixels per cell = 10 × 10 (size of a cell (in pixels)), number of orientation bins = 9, number of cells in each block = 2 × 2, and using the L2 block normalization method [57]. To improve the accuracy, we used background subtraction using motion analysis by applying BackgroundSubtractorMOG2 [58] (OpenCV) prior to the HOG extraction. The following parameters were used: history = 150 (number of last frames that affect the background model), varThreshold = 40 (variance threshold for the pixel-model match), and detectShadows = False.
- **Frame Vote**. We designed this algorithm to analyze each frame individually, using a different paradigm compared to the previous two algorithms. For each frame, we run a machine learning algorithm (HOG + SVM or CNN) that classifies the gesture. After 20 frames, the predicted gestures are compared and the one with the most occurrences is selected (majority vote). The algorithm is illustrated in Figure 3. The advantage of this method is that it does not take into account the sequence of the gesture, and thus may hold better if the sensing continuity is not entirely reliable, as may be the case in harsh environments. This is also a good fit for the continuous and repeated gestures elicited in the previous study, as the order in which the gestures are performed has less importance.

**Figure 4:**
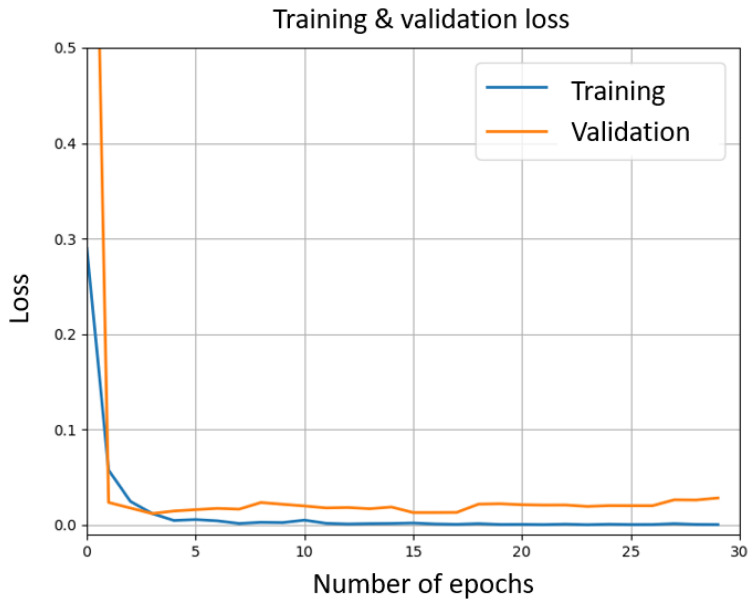
Training (blue) and validation (orange) loss through epochs for the CNN.

**Figure 5:**
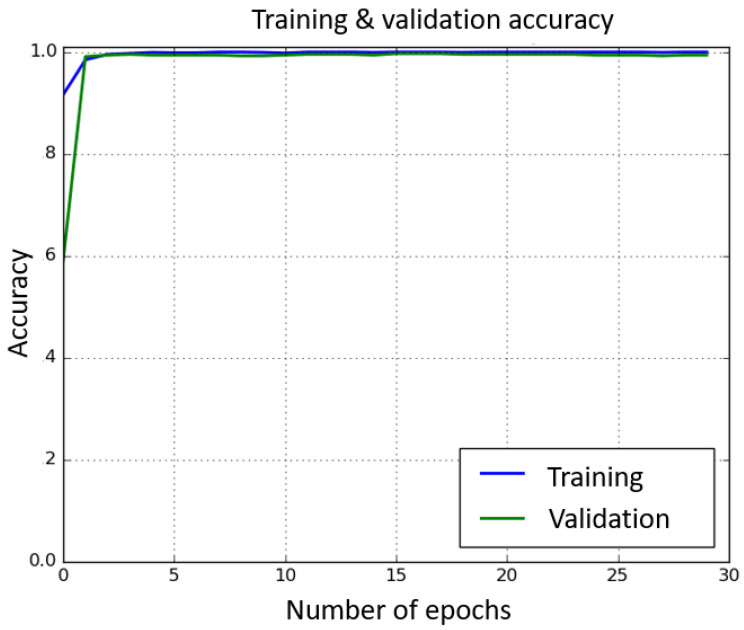
Training (blue) and validation (green) accuracy through epochs for the CNN.

The algorithm works as follows: a label (i.e., gesture) is predicted for each frame $\hat{y_{i}}$. Denoting by $n_{i}$ the number of frames labeled $\hat{y_{i}}$, we then assign the label $\hat{y}={\hat{y}}_{{\mathrm{argmax}}_{i}n_{i}}$ to the gesture. The HOG and SVM configurations are as described above. For CNN, we used the ResNet50V2 architecture from Keras API [59,60], as it provides a good trade-off between computation time and accuracy [45,61]. We loaded weights pre-trained on ImageNet [62]. The last layer of the model is with Softmax activation and 7 outputs (corresponding to 7 situations described in Section 4.3). The model is compiled using Keras with TensorFlow backend. We used categorical cross-entropy loss function and Stochastic Gradient Descent (SGD) optimizer with the default learning rate of 0.01. We split the overall amount of training data into 80% training and 20% validation, and trained the network with 10 epochs and with a batch size of 8. Training and validation loss decreased quickly and remained close to 0 after 10 epochs (respectively, 0.0028 and 0.0236) (see Figure 4). Training and validation accuracy reached 98.48% and 99.11%, respectively, after two epochs only, and after 10 epochs, training accuracy remained over 99.9%, while validation accuracy stayed stable at around 99.4% (see Figure 5).

### 4.3. Data Collection and Training

We collected data from 13 people (5 f, 8 m) from the research lab, who performed the four user-defined gestures. Each person was also asked to both walk and stand at various distances in front of the camera from a distance of 1.5 m to a distance of 5 m. Participants performed omrieach gestures in front of the sensors and the data collection was conducted across four different indoor locations. Lighting conditions for the four locations ranged between 100 and 700 lux. The distance from the participants to the background ranged between 0.2 and 4 m. The temperature for all locations was an ambient temperature of 25 °C to 28 °C.

We gathered and trained the system to recognize when there was no person in front of it, so that it could differentiate this situation from the others. We gathered data points as follows for each sensor: 13 people × 7 situations (4 user-defined gestures + walking + standing + no person) × 15 trials × 20 frames = 27,300 frames, which were then used to train the algorithms. As part of the data collection process, we measured the average completion time for the user-defined gestures across participants (for one repetition, in seconds): the longest duration is “Mapping” (μ=3.05,SD=0.65), followed by “Identifying Hazardous Materials” (μ=2.26,SD=0.7), “Finding Survivors” (μ=1.13,SD=0.27), and “Detecting Fire” (μ=1.04,SD=0.26).

Note that these data were collected under the baseline conditions (i.e., without firefighting equipment and without added smoke). Our idea was to train the system under regular conditions and then determine if, and to what extent, a system trained under regular conditions would perform over different types of harsh conditions.

### 4.4. Evaluation

**Figure 6:**
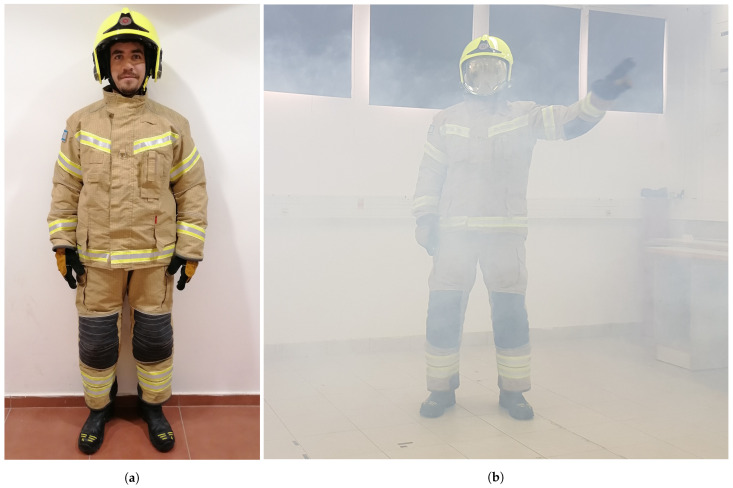
(**a**) Firefighting equipment including helmet, jacket, trousers, and gloves and (**b**) experiment conducted to evaluate the system’s accuracy with full equipment and smoke.

**Figure 7:**
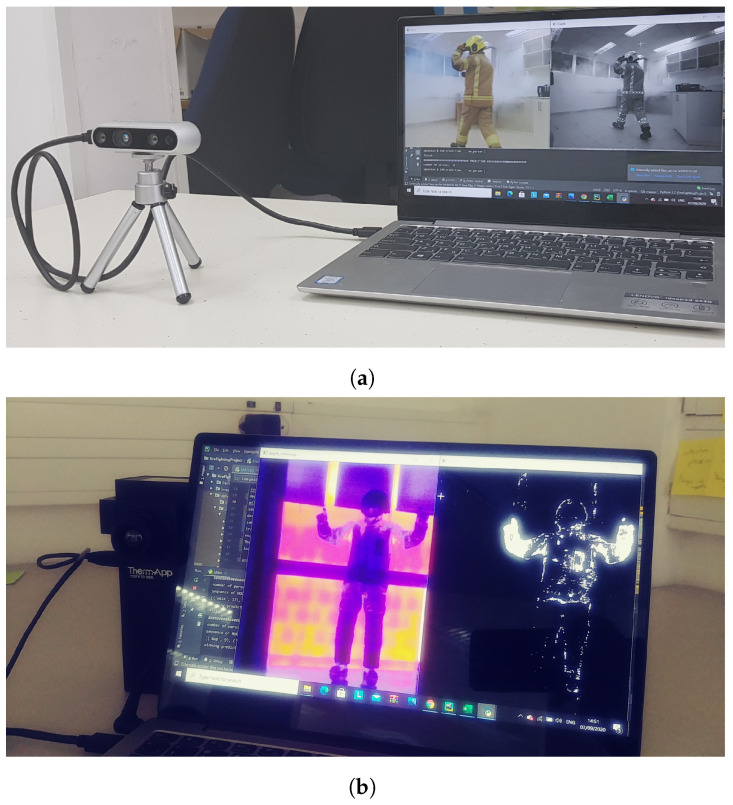
Examples of the setup used in the evaluation of our system. The person is wearing protective firefighting equipment and smoke is starting to be generated. (**a**) The Realsense camera feed is shown on the laptop. (**b**) The raw thermal sensor feed is shown on the left side of the laptop’s screen and, on the right side of the laptop’s screen, the feed is shown after background subtraction.

The aim of the evaluation phase was to evaluate how the sensors and algorithms would perform under different harsh conditions in real-time gesture recognition. The system was evaluated with two people (2 m) from the research lab who did not participate in the training phase. Each of them performed 6 situations (4 user-defined gestures + walk + stand) in front of the sensors. Each participant performed a total of 50 repetitions of the 6 situations in a random order (the ratio of situations tested was balanced). We further evaluated the system when no person was standing in front of the visual sensor. The evaluation was conducted under the following three conditions: 1. with the person wearing firefighting protective equipment, 2. with the person wearing firefighting protective equipment and with smoke in the room (Figure 6b), and 3. without equipment or smoke (i.e., baseline). The smoke was cold, dense smoke generated by a dedicated smoke machine in an indoor environment (derived from [7]), and all tests were run in controlled laboratory settings. Figure 7 illustrates the evaluation setup. Each of the 3 laboratory conditions was evaluated with all 3 sensors and the following algorithms: RGB (skeleton extraction + SVM), Depth (“Frame Vote” (HOG + SVM), “Frame Vote” (CNN), HOG + SVM), Thermal (“Frame Vote” (HOG + SVM), “Frame Vote” (CNN), HOG + SVM, skeleton extraction + SVM), resulting in a total of 24 settings.

The evaluation was conducted in conditions similar to the training phase. Lighting conditions were 500 lux, the distance between the participants and the sensors was 1.5 to 5 m, the distance from participants to the background ranged between 0.2 and 2 m, the participants performed the gestures in front of the sensors, and the ambient temperature was between 25 °C and 28 °C.

### 4.5. Results and Interpretation

We calculated the recognition accuracy as the ratio between the number of gestures correctly recognized over the total number of trials. These results are reported in Table 2. The accuracy was the highest overall with the skeleton extraction algorithm. When tested in baseline conditions (no smoke or equipment), we achieved, on the best algorithm, high accuracy for each sensor: 98% for RGB, 92% for depth, and 94% for thermal. With firefighting equipment on, the recognition accuracy remained high for RGB and thermal, with 96%, and lowered for depth, with 84%. When smoke was added, the recognition reached up to 90% with RGB and 88% with thermal but the depth sensor did not work in this condition. Although RGB performed slightly better than thermal, we know from the literature that its performance will degrade in darkness or under thicker smoke. We find that **these results are consistent with the literature about the effect of smoke on the three sensors, and that they hold in gesture recognition applications** [43].

In terms of algorithms, while the HOG + SVM technique achieved better accuracy than Frame Vote when tested without equipment and smoke, Frame Vote achieved better accuracy than HOG + SVM in smoke. Regarding the use of protective gear, the depth sensor was the most affected, but the other sensors’ recognition was not significantly affected. The skeleton extraction + SVM algorithm appeared the most robust to protective gear, with only a 2% decrease for the RGB sensor, and with an increase of 2% in accuracy for the thermal sensor. In addition, we have shown that skeleton extraction of key points can be done with thermal images, although the API was built for RGB data.

**Figure 8:**
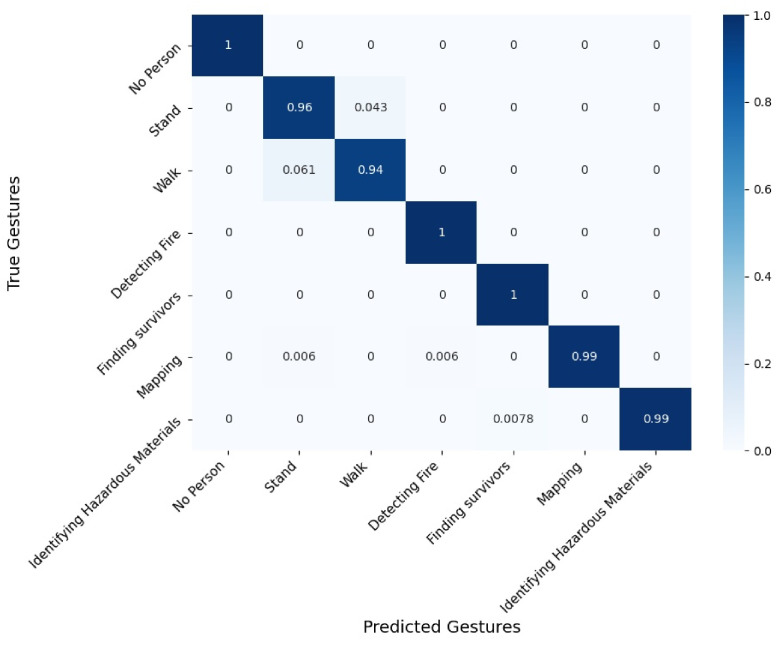
Confusion matrix for the RGB sensor with skeleton extraction + SVM, without smoke, and without equipment.

**Figure 9:**
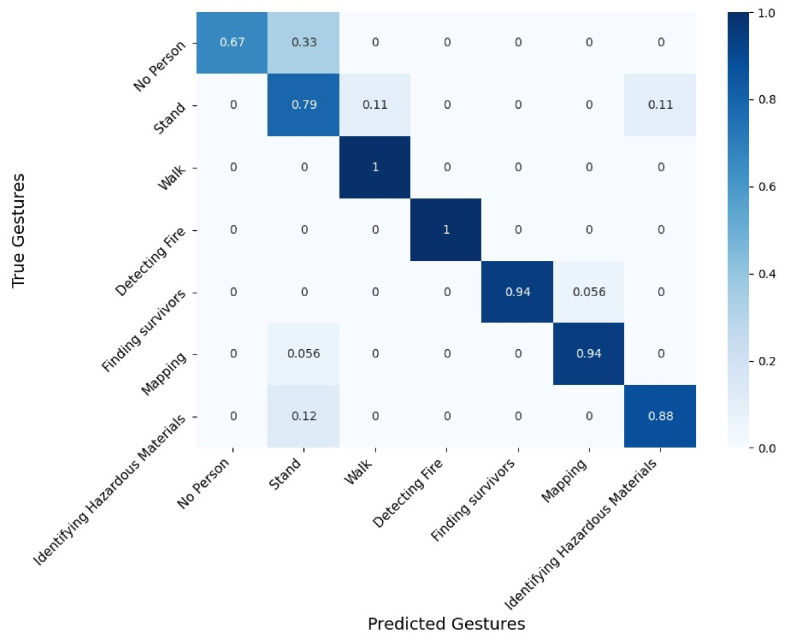
Confusion matrix for the depth sensor with HOG + SVM, without smoke, and without equipment.

**Figure 10:**
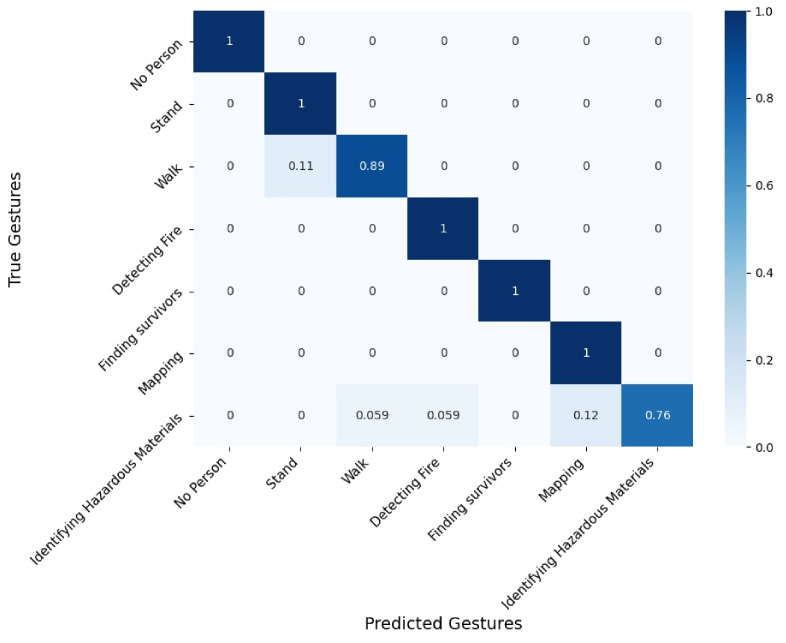
Confusion matrix for the thermal sensor with skeleton extraction + SVM, without smoke, and without equipment.

Figure 8, Figure 9 and Figure 10 present confusion matrices corresponding to 3 evaluated conditions out of the 24. We present the following three settings: RGB sensor with skeleton extraction, without smoke, and without equipment (Figure 8); depth sensor with HOG + SVM, without smoke, and without equipment (as in Figure 9); and thermal sensor with skeleton extraction + SVM, without smoke, and without equipment (as in Figure 10). The confusion matrices are used to demonstrate the recognition accuracy of each gesture and to describe the type of error made by the system.

Figure 8 presents the confusion matrix from the RGB sensor with skeleton extraction, without smoke, and without equipment, which shows an overall measured accuracy of 98%. We found that 3 out of 7 situations are recognized perfectly: “No Person”, “Detecting Fire”, and “Finding Survivors”. “Stand” was accurately recognized 96% of the time and was confused with “Walk” 4% of the time and “Walk” was accurately recognized 94% of the time and was confused with “Stand” 6% of the time. “Mapping” was accurately recognized 99% of the time and was confused with either “Stand” or “Detecting Fire” (<1% each). Finally, “Identifying Hazardous Materials” was accurately recognized 99% of the time and was confused with “Finding Survivors” (<1%).

Figure 9 presents the confusion matrix from the depth sensor with HOG + SVM, without smoke, and without equipment, which shows an overall measured accuracy of 92%. We found that 2 out of 7 situations are recognized perfectly: “Walk” and “Detecting Fire”. “No Person” was accurately recognized 67% of the time and was confused with “Stand” 33% of the time, while “Stand” was accurately recognized 79% of the time and was confused with “Walk” and “Identifying Hazardous Materials” (approximately 11% each). The major difference in the accuracy of these 2 situations, between this setting and RGB with skeleton extraction + SVM, can be explained by the fact that we used background subtraction based on motion analysis (see Section 4.2). When the participant is standing still, it appears as if there is no person in the scene after applying background subtraction. As such, this configuration introduced a confusion between the 2 situations, which further reveals a clear disadvantage of applying this background subtraction to static gestures. “Finding Survivors” was accurately recognized 94% of the time and was confused with “Mapping” 6% of the time. “Mapping” was accurately recognized 94% of the time and was confused with “Stand” 6% of the time. Finally, “Identifying Hazardous Materials” was accurately recognized 88% of the time and was confused with “Stand” 12% of the time.

Figure 10 presents the confusion matrix of the thermal sensor with skeleton extraction + SVM, without smoke, and without equipment, which shows an overall measured accuracy of 94%. In this condition, 5 situations out of 7 are recognized perfectly: “No person”, “Stand”, “Detecting Fire”, “Finding Survivors”, and “Mapping”. “Walk” was accurately recognized 89% of the time and was confused with “Stand” 11% of the time. This is not surprising given that standing corresponds to a subset of frames of walking. “Identifying Hazardous Materials” was the least recognized situation, with 76% accuracy, and was mainly confused with “Mapping” (12%) but also with “Detecting Fire” and “Walk” (5.9% each). When inspecting the user-defined gestures, we found similarities between the “Identifying Hazardous Materials” and “Mapping” gestures that present overlapping frames (when both hands are either in front of the face or moving from the side of the body to the face), which may explain the confusion between the two gestures.

Comparing these 3 confusion matrices, which all represent one laboratory setting (without smoke and without equipment), enables us to better understand some of the advantages and drawbacks of the algorithms or to identify differences and similarities in the gestures. For example, our findings suggest that the “Mapping”, “Detecting Fire”, and “Finding Survivors” gestures were overall successfully recognized and differentiated from the other gestures, with 94% to 100% accuracy in the presented settings. However, the “Identifying Hazardous Materials” gesture was recognized, with lower accuracy (76% to 99% in the presented settings), which suggests that this gesture might not be appropriate in the first response context, which is not tolerant to mistakes. It is important to note that when these gestures were elicited with firefighters, no algorithmic perspective was considered. However, as first responders are trained personnel, they can be taught specific gestures, and the “Identifying Hazardous Materials” could be modified in an iterative process with the firefighters to reduce its overlap with other gestures. In addition, we see that skeleton extraction + SVM achieved higher accuracy for the gesture “Identifying Hazardous Materials” with the RGB sensor (99%) than with the thermal sensor (76%), which can be explained by the fact that the API was trained with RGB data only. These findings highlight the need to train the skeleton extraction approach with additional types of sensor data for a wider range of needs.

## 5. Discussion

In this work, we compared how different remote visual sensors and algorithms perform under different conditions related to harsh environments. In terms of sensors, our results showed that the RGB camera performs relatively well in a smoky environment, although prior work showed that it is expected to fail in dense smoke when visibility decreases below 1 m. Depth sensors, on the contrary, which are designed to work well in darkness, did not allow for gesture recognition in smokey conditions, even with light smoke. The thermal sensor, which also performs well in darkness, maintained high accuracy in our experiment, even under smoky conditions and with the use of firefighting equipment. Based on our findings, and combined with the fact that thermal sensors can effectively detect fires [63], we propose that thermal sensors would be the best compromise for gestural recognition in harsh conditions. Nonetheless, we envision that future robotic systems will be required to perform well in both regular and harsh conditions, and in both daylight and darkness. As such, we propose that having multiple sensors and using sensor fusion algorithms would be a safer solution. While this solution should be easily adaptable on ground robots, weight limitations on aerial robots will probably still constrain researchers to a limited amount of sensors and computing power, for which we suggest that thermal sensors are most suitable.

However, thermal sensors also present limitations, such as high costs (over RGB and depth sensors). They are also less prevalent than RGB and depth cameras in existing robotic systems. Furthermore, RGB cameras typically provide higher-resolution images, while depth cameras provide the additional advantage that they can measure the distance between the robot and its environment, so that they can offer multiple usages. We therefore suggest that the decision regarding which sensor to embed on a robotic system shall take a holistic perspective in terms of the actual system’s needs.

In terms of algorithms for gestural recognition in harsh environments, we found that the skeleton extraction + SVM approach provided good results (with up to 98% accuracy in baseline conditions and 90% accuracy in smoke and with equipment), even though the system was not trained in harsh environment settings. Since there are no publicly available relevant datasets with gestures adapted to the firefighting context, we propose to compare our results to prior work using similar settings. For example, when comparing the results from the RGB sensor with skeleton extraction + SVM, without equipment, and without smoke, to recent results from Liu and Szirányi [54] in similar settings with skeleton extraction (OpenPose) + DNN, we find that our results are on par with the state-of-the-art. Indeed, their model can recognize 10 body gestures at 99.8% accuracy using their test set, against 98% in our system. While they achieved 1.8% higher accuracy, only 2 of their 10 gestures were dynamic, the rest being static. This may then explain the small difference in accuracy; however, further research should specifically compare static vs. dynamic gestures.

One of the goals of our experiment was to verify the effect of the three harsh environmental conditions on the sensors and algorithms’ performances. We have shown that, although the system was trained in normal conditions (i.e., without smoke and equipment), the presence of protective equipment on the person did not affect the RGB and thermal sensors’ recognition rates, and that the skeleton extraction + SVM was the least affected method. Moreover, when smoke was added, all three sensors suffered from a decrease in accuracy, with skeleton extraction + SVM being, once again, the least affected method.

## 6. Limitations and Future Work

This work investigated the performance of three common sensors for gesture recognition in simulated harsh environments. While we found interesting differences between the sensors, future work should consider additional sensors operating at different wavelengths or using different technology, as recent work compared LIDAR perfomance in smoke to six other sensors [43]. Our evaluation considered different harsh environments simulated in an indoor controlled laboratory setup. In one setting, we generated cold and dense smoke with a dedicated machine. Future research could vary the density and temperature of the smoke (e.g., light smoke with a high temperature [43]) and consider smoke generated from various burning materials. Future research could further expand to other algorithms, such as by using RNN or LSTM for the classification of gestures, or by expanding the “Frame Vote” algorithm by introducing weights to the frames.

In our work, we chose to test the gesture recognition system using a fixed setup, before embedding the sensor and computation on a robot or a drone, as per prior work [37,50]. As such, future work will investigate additional constraints related to embedding the system and testing it in real-world conditions that will affect the recognition accuracy. These include environmental factors such as various types of lighting (indoors or outdoors), different orientations and angles between people and sensor, managing jitter, evaluating the effect of occlusion due to objects such as debris, and considering various heat sources in the scene, since the performance of thermal sensors will be affected by them (e.g., open fire) [64].

We further expect that when training the system with data collected in additional environmental settings, we will see a lesser decrease in the accuracy between the baseline (i.e., no equipment or smoke) and harsh conditions (e.g., equipment and smoke). Finally, future research should implement the provided methods and conduct empirical research with first responders in simulated or real harsh conditions.

## 7. Conclusions

This manuscript presented research into gestural recognition in harsh environments tailored for first responders. In particular, we investigated appropriate sensors and algorithms for the remote sensing of body gestures from ground or aerial robot. We designed and prototyped a gestural interaction system using three types of sensors, namely RGB, depth, and thermal, and a series of algorithms and feature extraction techniques. We trained the system (N = 13) and evaluated it (N = 2) in three simulated harsh environments. Our results highlight differences between sensors and algorithms that can inform future researchers and practitioners on the human and technical aspects to consider when integrating gestural recognition systems in harsh environments.

## Figures and Tables

**Table 1:** Drone tasks used as referents for the elicitation study. The corresponding user-defined gestures are presented in Figure 1.

Task	Description	Chosen Gesture
Detecting Fire	Detect number and location of fire spots	(a)
Identifying Hazardous Materials	Identify source and size of gas or chemical leak	(b)
Finding Survivors	Search for trapped citizens or people in danger and collect information on their number and location	(c)
Mapping	Create a map of the floor or building based on exploration	(d)

**Table 2:** Recognition accuracy results for each sensor and algorithm evaluated (see Section 4.4).

		Recognition Accuracy		
Sensor	Algorithm	without Smoke without Equipment	without Smoke with Equipment	with Smoke with Equipment
Depth	“Frame Vote” (HOG + SVM)	78%	70%	N/A
	“Frame Vote” (CNN)	81%	72%	N/A
	HOG + SVM	92%	84%	N/A
RGB	skeleton extraction + SVM	98%	96%	90%
Thermal	“Frame Vote” (HOG + SVM)	90%	86%	70%
	“Frame Vote” (CNN)	90%	84%	71%
	HOG + SVM	86%	86%	56%
	skeleton extraction + SVM	94%	96%	88%

## Data Availability

The data presented in this study are available on request from the corresponding author.

## Abbreviations

The following abbreviations are used in this manuscript:

HRI	Human–robot interaction
HCI	Human–computer interaction
HDI	Human–drone interaction
SVM	Support vector machine
HOG	Histogram of oriented gradient
CNN	Convolutional neural networks
RNN	Recurrent neural network
LSTM	Long short-term memory
